# Disability and health-related quality of life in patients undergoing spinal fusion: a comparison with a general population sample

**DOI:** 10.1186/1471-2474-14-211

**Published:** 2013-07-17

**Authors:** Liisa Pekkanen, Marko H Neva, Hannu Kautiainen, Joost Dekker, Kirsi Piitulainen, Marko Wahlman, Arja Häkkinen

**Affiliations:** 1 Department of Orthopaedics and Traumatology, Jyväskylä Central Hospital, Jyväskylä, Finland; 2 Department of Orthopaedics and Traumatology, Tampere University Hospital, Tampere, Finland; 3 Unit of Family Practice, Central Finland Central Hospital, Jyväskylä, Finland; 4 Unit of Primary Health Care, Kuopio University Hospital, Kuopio, Finland; 5 VU Medical Center Amsterdam, Amsterdam, Netherlands; 6 Department of Physical Medicine and Rehabilitation, Jyväskylä Central Hospital, Jyväskylä, Finland; 7 Department of Health Sciences, University of Jyväskylä, Jyväskylä, Finland

**Keywords:** Spinal fusion, Oswestry disability index, Health-related quality of life, General population sample

## Abstract

**Background:**

The aim of the present study was to compare one-year-follow-up data on disability and health-related quality of life (HRQoL) between spinal fusion patients and age- and sex-matched general population.

**Methods:**

The data on fusion patients were collected prospectively using a spinal fusion data base in two Finnish hospitals. A general population sample matched for age, sex and residential area was drawn from the Finnish Population Register. All participants completed a questionnaire and the main outcome measures were the Oswestry Disability Index (ODI) and the Short Form-36 questionnaire (SF-36).

**Results:**

Altogether 252 (69% females) fusion patients and 682 (67% females) population sample subjects participated in the study. In general population the mean ODI was 15 (SD 17) in females and 9 (SD 13) in males. The corresponding preoperative ODI values were 47 (SD16) and 40 (SD 15) and one year follow-up values 22 (SD 17) and 23 (SD 20). In both sexes the ODI decreased significantly after surgery but remained higher than in the general population, p < 0.001. The physical component summary score (PCS) of the SF-36 was lower in the patients than general population sample both preoperatively and at one-year follow-up (p < 0.001). The mental component summary score (MCS) was lower preoperatively (p < 0.001), but reached the general population level after one year in both men (p = 0.42) and women (p = 0.61).

**Conclusions:**

Disability and HRQoL improved significantly after spinal fusion surgery during a one- year follow-up. However, the patients did not reach the level of the general population in the ODI or in the physical component of HRQoL at that time, although in the mental component the difference disappeared.

## Background

With the ageing of the population, an increase in degenerative spine conditions and the number of surgical patients can be expected [1]. Although instrumented spinal fusions have been performed since the early 1960s, these procedures remain controversial owing to inconsistent responses to the treatment [2,3]. In the field of spinal fusion outcome research, most earlier trials have compared surgical methods and assessed the success of the surgical procedure itself. However, over the last few decades there has been a trend towards the use of patient-reported outcomes (PROs) in evaluating the outcome of fusion surgery in addition to physical examinations, imaging or clinical outcome scales. Recently, the routine administration of certain instruments in connection with low back pain and surgical treatment has been recommended [4,5]. Condition-specific disability measures like the Oswestry Disability Index (ODI) should be used before and after surgical treatments. When evaluating surgical outcomes in the clinical-research setting, Health Related Quality of Life (HRQoL) tools, such as the Short-Form 36 (SF-36), Short Form 12 (SF-12) or EuroQol Group (EQ-5D) should be used [4].

Information on whether the operation provides effective relief of symptoms and disability continues to be lacking [3]. In defining success after spinal fusion operations, calculation of the minimum clinically important difference (MCID) in PROs has been suggested. This method has limitations; for example the MCID values may differ according to multiple factors, such as original spine pathology, the method of treatment, sample size and patient-characteristics, e.g. baseline scores [6]. Another method that has been proposed is based on prospective minimum goals established by individual patients themselves. In the study by Carragee et al. isthmic spondylolisthesis patients and degenerative disc disease patients preoperatively indicated their expectations concerning level of function (ODI), work capacity, pain intensity and medication requirement [7]. One of most recent attempts to solve the difficulty in defining clinical success after spinal fusion operations is to analyze whether the patients reach the level of the general population in certain PROs. To our knowledge only one study by Mokhtar et al. [1]. has used this method. Prospective data on 100 patients undergoing spinal fusion were collected using the SF-12 questionnaire, and the results were compared to those obtained for a sample of the general population. No disease-spesific disability measurement was used in this study. As only limited amount of information exists on the use of this method, so there is a clear need for further studies.

The aim of the present study was to compare disability and HRQoL among spinal fusion patients within one-year follow-up with the values of an age- and sex-matched population resident in the same district.

## Methods

Since the beginning of 2008, all patients undergoing spinal fusion surgery in Tampere University Hospital or Jyväskylä Central Hospital have been recruited to a prospective follow-up study.

In August 2010, the spinal database comprised 285 patients with the 6 most common diagnoses for elective spinal fusion. These diagnoses were degenerative spondylolisthesis, spondylolysis, spinal stenosis, disc herniation or degeneration, postoperative conditions and degenerative scoliosis. Disability and HRQoL measures were available preoperatively and 3 and 12 months postoperatively for 252 of these patients (88%) all of whom were included in this study. Six surgeons had performed the operations, and in most cases in teams of two surgeons.

The cohort of spinal fusion patients was compared to a general population sample matched according to age, sex and residential area. Four controls for each of these fusion patients was drawn from the Finnish Population Register and the sampling was performed by the Statistics Finland. A questionnaire was mailed to 1 140 controls in September 2010, and one reminder letter was sent two months later. After one reminder letter, the percentage of returned answers was 61% (n = 691) and the number of acceptable answers 682.

One to two weeks prior to the fusion operation, the patients filled in a questionnaire requesting sociodemographic and clinical information, for example weight, height, presence of co-morbidities, exercise habits, smoking and employment status. The main outcome measures were the Oswestry Disability Index (ODI) and the Short Form-36 Questionnaire (SF-36). The ODI is one of most widely used back-specific disability measurement tools in both clinical work and research.[8,9] According to the original publication, the scores are grouped into five categories: 0–20 minimal, 20–40 moderate, 40–60 severe disability; 60–80 crippled and 80–100 indicates that the patient is either bed-bound or exaggerating his or her symptoms [8]. The Finnish validated version 2.0 of the ODI was used [10]. The SF-36 is a generic patient-assessed health outcome measure for health-related quality of life with eight dimensions reflecting patients’ health and welfare. The SF-36 score also divides into two summary measures: the physical component summary score (PCS) and the mental component summary score (MCS). The dimensions Physical Functioning, Role Physical, Bodily Pain and General Health form the PCS, and Mental Health, Vitality, Social Functioning and Role-Emotional the MCS. Version 1 of the SF-36 qustionnaire was used on this study.

The ethical committees in Tampere University Hospital and Jyväskylä Central Hospital approved the study plan and all the participating patients signed a written consent.

### Statistics

Results are expressed as mean and standard deviation (SD). Statistical comparison between the groups was performed by t-test, bootstrap-type t-test (5000 replications), or chi-square test, where appropriate. Differences in the ODI and HRQoL between the groups were determined using generalized linear models. Repeated measures were analyzed using linear mixed models.

## Results

The demographical and clinical data of the fusion patients and general population is shown in Table 1. Sixty-nine per cent of the 252 fusion patients and 67% of the 682 general population subjects were females. In the population sample, the mean age of females was higher than in the patient group: 66 (SD 11) vs. 63 (SD 12) years (p = 0.014). The mean age of males was 60 (SD 13) years in the general population and 58 years in the patients (p = 0.43). In both sexes the body mass index (BMI) was significantly higher in the fusion patients than in the general population. The number of cardiological (p < 0.001) and rheumatoid co-morbidities (p = 0.012) was higher in the female patients than in the female population subjects and the female patients were also less physically active. In the general population, 5.7% of the females and 8.0% of the males had spinal disorders.

**Table 1:** Demographical and clinical data

**Variables**	**Female**		**p-value**	**Male**		**p-value**
	**Patients**	**Population**		**Patients**	**Population**	
	**n = 174**	**n = 458**		**n = 78**	**n = 224**	
Body mass index, mean (SD)	28.1 (4.5)	26.9 (4.7)	0.0046	28.0 (3.8)	26.8 (3.8)	0.021
Co-morbidities, n (%)						
Cardiological	100 (59)	197 (43)	<0.001	37 (48)	81 (36)	0.065
Respiratory	24 (14)	51 (11)	0.29	4 (5)	15 (7)	0.64
Neurological	7 (4)	26 (6)	0.45	2 (3)	10 (4)	0.47
Rheumatoid	21 (12)	29 (6)	0.012	2 (3)	3 (1)	0.46
Diabetes	15 (9)	60 (13)	0.15	14 (18)	27 (12)	0.18
Psychiatric	7 (4)	18 (4)	0.90	2 (3)	7 (3)	0.82
Musculosceletal	10 (6)	48 (10)	0.080	1 (1)	7 (3)	0.39
Education, years, mean (SD)	11.3 (3.5)	11.6 (4.2)	0.33	11.5 (3.4)	11.7 (3.7)	0.78
Tobacco use, n (%)	17 (10)	46 (10)	0.99	13 (17)	42 (19)	0.72
Employment situation, n (%)			0.71			0.38
Employed	50 (29)	140 (31)		37 (47)	98 (44)	
Unemployd	4 (2)	15 (3)		2 (3)	15 (7)	
Retired	120 (69)	303 (66)		39 (50)	111 (49)	
Leisure time physical activity hours per week, mean (SD)	3.3 (3.7)	4.4 (3.9)	0.0019	4.7 (4.2)	4.6 (6.2)	0.95

**Figure 1:**
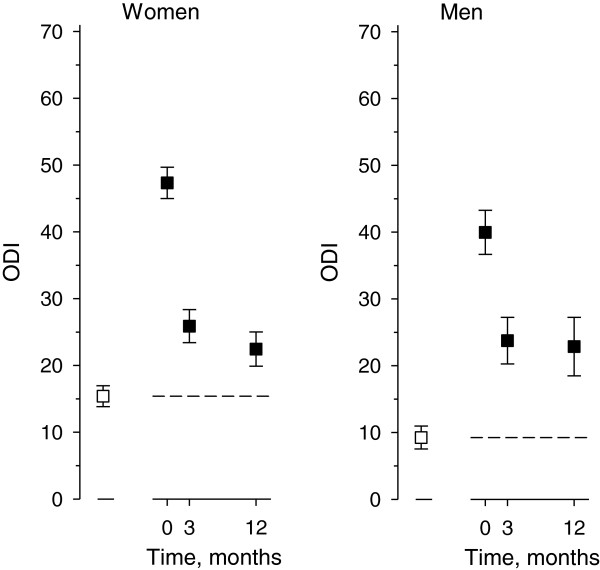
The mean Oswestry Disability Index (ODI, with 95% Confidence Interval ) in the patients (■ ) and in the population ( □, dashed line).

In the general population, females had higher ODI (15 (SD17)) than males (9 (SD 13)), (p < 0.001). In the patients, the preoperative ODI values were 47 (SD16) in females and 40 (SD 15) in males (p < 0.001) (Figure 1). One year post fusion the mean change in ODI was −25 (95% CI −28 to −22) in females and −17 (95% CI −21 to −13) in males. However, in both sexes, the age-adjusted ODI scores at baseline and at one-year were significantly higher than the mean ODI values in the general population (p < 0.001). The postoperative change in ODI between three months and one year was minor and not significant in males while in females the change was significant.

All the SF-36 dimensions in the general population were significantly better than the preoperative values of the patients, both in females and males, (p < 0.001). (Table 2). In both sexes the preoperative mean ratio between the patients and the general population subjects was biggest in the dimension Role-Physical. At the one-year follow-up the female patients reached the population level in Vitality, Mental Health and Role-Emotional, while male patients reached the population level only in Vitality and Mental Health.

**Table 2:** Health-related quality of life in population and patients preoperatively stratified by sex

	**Population**	**Patients**		Mean ratio* (95% CI)			
**SF-36 dimensions**	**Mean (SD)**	**Preoperative mean (SD)**	**12 months mean (SD)**	**Preoperative**	**p-value patients vs population preoperative**	**12 months**	**p-value patients vs population 12 months**
Female							
Physical functioning	70 (28)	28 (19)	58 (29)	2.6 (2.3 to 3.0)	<0.001	1.3 (1.2 to 1.4)	<0.001
General health	60 (22)	53 (20)	56 (21)	1.1 (1.1 to 1.2)	<0.001	1.1 (1.0 to 1.1)	0.022
Vitality	65 (23)	45 (22)	64 (23)	1.5 (1.3 to 1.6)	<0.001	1.0 (1.0 to 1.1)	0.41
Mental health	77 (19)	63 (21)	77 (19)	1.2 (1.2 to 1.3)	<0.001	1.0 (1.0 to 1.0)	0.79
Role physical	64 (42)	9 (21)	44 (43)	7.9 (2.7 to 13.0)	<0.001	1.5 (1.3 to 1.7)	<0.001
Role emotional	71 (39)	46 (43)	67 (41)	1.6 (1.4 to 1.8)	<0.001	1.1 (1.0 to 1.2)	0.17
Social functioning	82 (25)	46 (28)	76 (28)	1.8 (1.7 to 2.0)	<0.001	1.1 (1.0 to 1.1)	0.004
Bodily pain	67 (27)	24 (15)	56 (25)	2.8 (2.4 to 3.3)	<0.001	1.2 (1.1 to 1.3)	<0.001
Male							
Physical functioning	84 (22)	39 (20)	62 (26)	2.2 (1.9 to 2.4)	<0.001	1.3 (1.2 to 1.5)	<0.001
General health	65 (21)	56 (21)	55 (23)	1.2 (1.1 to 1.3)	<0.001	1.2 (1.1 to 1.3)	<0.001
Vitality	71 (22)	53 (23)	66 (24)	1.4 (1.2 to 1.5)	<0.001	1.1 (1.0 to 1.2)	0.072
Mental health	81 (18)	68 (21)	76 (19)	1.2 (1.1 to 1.3)	<0.001	1.1 (1.0 to 1.2)	0.074
Role physical	74 (38)	12 (21)	44 (43)	6.3 (2.1 to 10.5)	<0.001	1.7 (1.3 to 2.0)	<0.001
Role emotional	79 (35)	44 (43)	65 (42)	1.8 (1.5 to 2.2)	<0.001	1.2 (1.1 to 1.4)	0.003
Social functioning	87 (20)	62 (27)	75 (36)	1.4 (1.3 to 1.5)	<0.001	1.2 (1.1 to 1.3)	<0.001
Bodily pain	75 (23)	30 (16)	55 (29)	2.5 (2.1 to 3.0)	<0.001	1.4 (1.2 to 1.5)	<0.001

*Adjusted age.

**Figure 2:**
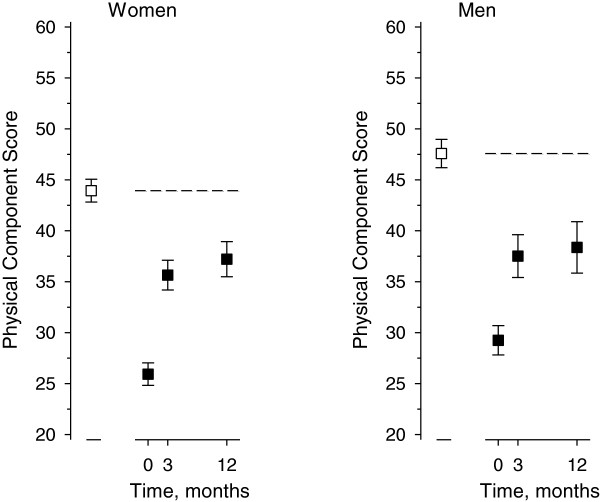
**The change in the Physical Component Summary Score of SF-36 in patients ( ■) compared with the population sample ( □, dashed line).** Results are mean with 95% Confidence Interval.

In the general population, the PCS of the SF-36 was 44 (SD 11) in females and 48 (SD 10) in males (Figure 2). Among the patients the preoperative PCS was 26 (SD 7) in females and 29 (SD 6) in males. At 12 months post surgery, the change in the PCS was 11 (95% CI 10 to 13; p < 0.001) in females and 10 (95% CI 7 to 12; p < 0.001) in males.

**Figure 3:**
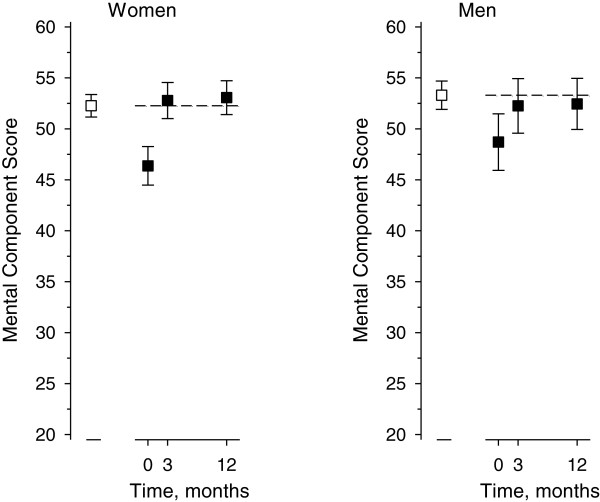
**The change in the Mental Component Summary Score of SF-36 in patients (■ ) compared with the population sample ( □, dashed line).** Results are mean with 95% Confidence Interval.

In turn the MCS of the SF-36 was 52 (SD 11) in females and 53 (SD 10) in males in the general population. The preoperative MCS was 46 (SD 13) in the female patients and 48 (SD 12) in the male patients. The positive change in the MCS from the preoperative to 12-month values was 7 (95% CI 5 to 8; p < 0.001) in females and 4 (95% CI 1 to 6; p < 0.001) in males (Figure 3). In the MCS, both the female (p = 0.42) and male (p = 0.61) patients had reached the level of the general population at one year post surgery, although, the difference in PCS between the patients and the general population remained significant (both sexes p < 0.001). In the patients, the changes in PCS and MCS between three months and one year after surgery, were minor and statistically non significant.

## Discussion

Our main purpose was to study the recovery of the spinal fusion patients during a one-year follow-up and compare our patients reported outcomes (PRO) to the values of a matched general population sample. The results showed that despite considerable improvement during the follow-up the patients did not reach the level of the matched general population in either disability or the physical component of the HRQoL.

To our knowledge, this is the first study where the PROs of spinal fusion patients have been compared to general population values. The general population subjects showed minimal disability in the mean ODI scores according to the original scoring, while the fusion patients’ mean ODI scores were preoperatively severe and at one year after the spinal fusion surgery remained moderate [8]. Therefore, inspite of recovery the disability according to the ODI did not decrease to the level of general population in our follow-up in males or in females. One explanation for this might be, that the patients undergoing fusion operation have often suffered from longstanding spinal symptoms which may have caused permanent changes to their life and behavior.

Interestingly the change in the ODI between 3 months and one year was minimal. This finding suggests that already the early recovery at three months may probably have quite high prognostic value when assessing the success of the treatment, also over a longer period. This result is supported by a finding in the earlier literature [11]. In a study of 96 patients undergoing spinal fusion, pain measurements were conducted at 6 months and then yearly over a total follow-up of 5 years. An interesting finding was that the improvement in the pain scale was biggest at 6 months and in the ODI at one year. The improvements seen in this early phase were maintained throughout the remainder of the follow-up period [11].

In the present study, one of the main findings concerning disability was that the mean levels of the ODI had not reached the values of the general population in either sex at one year post surgery. In comparison with the results in disability reported in the literature, in a trial implemented at 5 spine centers with 497 patients receiving one or two level spinal fusion, the mean ODI improved by 22 points at one year postoperatively. The preoperative level of the ODI varied in different subgroups from 48 to 56 [12]. In the Swedish Lumbar Spine Study, which was a multicenter randomized controlled trial where patients were randomized into a surgical or a control group, 222 patients received spinal fusion either by non-instrumented fusion, by instrumented posterolateral fusion or by circumferential fusion. In the surgical group, the mean ODI improved from 47 to 36 (p < 0.0001), at two-year follow up [13]. In a prospective randomized controlled study of 111 patients with adult isthmic spondylolisthesis the preoperative ODI scores were not reported but at two years in the surgical group the mean ODI score was 26 (95% CI 18.1 to 31.6) [14]. In our study at one year the mean positive change of the ODI in female patients was 25 (95% CI 22 to 28) and in male patients 17 (95% CI 13 to 21) and the corresponding mean ODI scores were 22 (SD 17) and 23 (SD 20).

In the present study, the spinal fusion patients reached the values of their matched population sample in the mental component (MCS) of SF-36 but not in the physical component (PCS). Preoperatively, the value of Role Physical was highest in patients in both sexes in the mean ratio analysis. At 12 months, Vitality, Mental Health and Role-Emotional were the only dimensions in the female patients that reached general population values. In males, this was true only for Vitality and Mental Health. Interestingly, the Pain dimension was still significantly worse in patients at the one-year follow-up compared to the general population. This prompts the question: how should we manage the physical aspect in the long term recovery. The earlier literature includes a multicenter study with 497 patients undergoing one- or two-level spinal fusion with several techniques. The results showed an improvement in mean PCS of 9.9 points over a one-year follow up [12]. This finding is confirmed by our study in which the mean PCS improved by 11 (95% CI 10 to 13) points in females and 10 (95% CI 7 to 12) in males in one-year follow-up. Another study with 100 primary spinal fusion patients who received decompression and single-level posterior lumbar interbody fusion reported HRQoL scores in both the PCS-12 and MCS-12 that approached the Australian population norm over a follow-up varying from 12 months to 5 years [1]. The mean postoperative PCS-12 score was 39 (95% CI 37 to 42) and MCS-12 score 52 (95% CI 50 to 55) as compared with the corresponding population norm values of 44 (95% CI 43 to 46) and 54 (95% CI 53 to 55) [1]. To our knowledge no other studies have used a population-based method when exploring the success of fusion operations.

After spinal fusion operations it is seldom a realistic goal to expect that all of the disability will disappear. It is also obvious that the level of disability, and hence quality of life, depends on various factors such as patient’s age, possible chronic neuropathic pain, and other possible diseases in addition to spinal disorders. However, in the present study, the prevalence of diabetes or most of the other co-morbidities, was similar in patients and in the general population. Interestingly only cardiovascular and rheumatoid diseases in females were more often present in patients than in the general population. Patients who have undergone spinal fusion may also get other sources of pain like osteoarthritis of hip or knee and these reasons may confuse the answers in the questionnaires. Furthermore, in spinal fusion surgery, complications and failed fusions may worsen the results. Finally, in the evaluation of disability of the patients it is essential to understand the level of disability in the general population of same age and sex. This data is important in evaluating the influence of surgery for the patients and also in surgical decision making in individual cases.

### Study strengths and limitations

The present study includes register based, not selected, consecutive patient material. The main strength of this study is the comparison between patients and the general population in disability and HRQoL scores. To our knowledge, this is the only study in which the PROs of fusion patients have been compared to those of a matched general population sample. An additional strength is the accurate timing of the data collection. The preoperative data were collected one to two weeks prior to the operation and the data collection timepoints during the follow-up were strict. In addition, the population based data were collected from the same residential area compared to the patients. In the analyses, females and males have been systematically stratified. This is because the majority of the patients operated on were females and because there was a significant gender difference in the ODI in the general population between females and males. A limitation in this study is the lack of analyses stratified by surgical diagnostic indication for the fusion operation. This is due the number of patients in this material, which could have led to a too small sample size in some of the diagnostic subgroups and lack of statistical representation of the phenomenon. 3Another limitation is that as a part of the surgical procedure in our patients, also decompression through laminectomy was performed whenever appropriate. This might cause difficulty to determine how much of the total improvement of HRQoL is caused by the fusion alone and how much by the coexisting decompression procedure. Further, a limitation is also the possible bias in answering to the general population questionnaire. Would those general population individuals who have back pain, reply more eagerly, making the observed difference between general population and patients smaller than the true value? In the literature it has been shown, that the life-time prevalence of back-pain in normal population is even 84% [15]. This leads to thinking, that even though there might be a bias in answering profile, it is not affecting the results between the patients and general population significantly. The follow-up in our study was 12 months. This period of time seemed to be sufficient to show, that results in disability and quality of life stabilized after three months. Although a one-year of follow-up indicated a trend towards recovery, further follow-ups of several years are needed to evaluate the longer term outcome.

## Conclusions

In this study the data of 252 spine fusion patients was analyzed and compared with general population. Despite the significant improvement during the one-year follow-up in both disability and HRQoL, the patients did not reach the level of general population in the ODI or in the PCS. In the MCS, however, both female and male patients reached the level of general population.

## Abbreviations

CI: Confidence interval; EQ-5D: The EuroQol group-5D; HRQol: Health-related quality of life; MCS: Mental component summary score; ODI: Oswestry disability index; PCS: Physical component summary score; PRO: Patient-reported outcome; SD: Standard deviation; SF-36: Short-form-36.

## Competing interests

The authors declare that they have no competing interests.

## Authors’ contributions

LP: corresponding author,participated in the design of the study, participated in the patient collection, drafted the manuscript, finished the manuscript, read and approved the final manuscript. MHN: participated in the design of the study, participated in the patient collection, revised the manuscript critically, read and approved the final manuscript. HK: performed the statistical analysis, revised the manuscript critically, read and approved the final manuscript. JD: participated in the design of the study, read and approved the final manuscript. KP: participated in to the patient collection, read and approved the final manuscript. MW: participated in the patient collection, read and approved the final manuscrip. AH: participated in the design of the study, helped to draft the manuscript, revised the manuscript critically, read and approved the final manuscript

## Pre-publication history

The pre-publication history for this paper can be accessed here:

http://www.biomedcentral.com/1471-2474/14/211/prepub

## References

[B1] Mokhtar SA McCombe PF Williamson OD Morgan MK White GJ Sears WR Health-related quality of life: a comparison of outcomes after lumbar fusion for degenerative spondylolisthesis with large joint replacement surgery and population norms Spine J 2010 10 4 306 312 10.1016/j.spinee.2010.01.018 20362246

[B2] Roy-Camille R Saillant G Mazel C Internal fixation of the lumbar spine with pedicle screw plating Clin Orthop Relat Res 1986 203 203 7 17 3955999

[B3] Gibson JN Waddell G Surgery for degenerative lumbar spondylosis: updated cochrane review Spine (Phila Pa 1976) 2005 30 20 2312 2320 10.1097/01.brs.0000182315.88558.9c 16227895

[B4] DeVine J Norvell DC Ecker E Fourney DR Vaccaro A Wang J Andersson G Evaluating the correlation and responsiveness of patient-reported pain with function and quality-of-life outcomes after spine surgery Spine (Phila Pa 1976) 2011 36 21 Suppl S69 S74 21897347 10.1097/BRS.0b013e31822ef6de

[B5] Chapman JR Norvell DC Hermsmeyer JT Bransford RJ DeVine J McGirt MJ Lee MJ Evaluating common outcomes for measuring treatment success for chronic low back pain Spine (Phila Pa 1976) 2011 36 21 Suppl S54 S68 21952190 10.1097/BRS.0b013e31822ef74d

[B6] Copay AG Glassman SD Subach BR Berven S Schuler TC Carreon LY Minimum clinically important difference in lumbar spine surgery patients: a choice of methods using the oswestry disability index, medical outcomes study questionnaire short form 36, and pain scales Spine J 2008 8 6 968 974 10.1016/j.spinee.2007.11.006 18201937

[B7] Carragee EJ Cheng I Minimum acceptable outcomes after lumbar spinal fusion Spine J 2010 10 4 313 320 10.1016/j.spinee.2010.02.001 20362247

[B8] Fairbank JC Couper J Davies JB O'Brien JP The oswestry low back pain disability questionnaire Physiotherapy 1980 66 8 271 273 6450426

[B9] Fairbank JC Pynsent PB The oswestry disability index Spine 2000 25 22 2940 2952 discussion 2952 10.1097/00007632-200011150-00017 11074683

[B10] Pekkanen L Kautiainen H Ylinen J Salo P Hakkinen A Reliability and validity study of the finnish version 2.0 Of the oswestry disability index Spine (Phila Pa 1976) 2011 36 4 332 338 20823785 10.1097/BRS.0b013e3181cdd702

[B11] Glassman SD Polly DW Dimar JR Carreon LY The cost effectiveness of single-level instrumented posterolateral lumbar fusion at 5 years after surgery Spine (Phila Pa 1976) 2012 37 9 769 774 10.1097/BRS.0b013e3181e03099 20489676

[B12] Glassman S Gornet MF Branch C Polly D Jr Peloza J Schwender JD Carreon L MOS short form 36 and oswestry disability index outcomes in lumbar fusion: a multicenter experience Spine J 2006 6 1 21 26 10.1016/j.spinee.2005.09.004 16413443

[B13] Fritzell P Hagg O Wessberg P Nordwall A Group SLSS Volvo award winner in clinical studies: lumbar fusion versus nonsurgical treatment for chronic low back pain: a multicenter randomized controlled trial from the swedish lumbar spine study group Spine (Phila Pa 1976) 2001 2001 26 23 2521 2532 discussion 2532–4 10.1097/00007632-200112010-00002 11725230

[B14] Ekman P Moller H Hedlund R The long-term effect of posterolateral fusion in adult isthmic spondylolisthesis: a randomized controlled study Spine J 2005 5 1 36 44 10.1016/j.spinee.2004.05.249 15653083

[B15] Walker BF The prevalence of low back pain: a systematic review of the literature from 1966 to 1998 J Spinal Disord 2000 13 3 205 217 10.1097/00002517-200006000-00003 10872758

